# A role of protein conformational dynamics in mammalian DNA methylation

**DOI:** 10.1063/4.0001204

**Published:** 2026-04-24

**Authors:** Jikui Song

**Affiliations:** Department of Biochemistry, University of California Riverside, Riverside, California 92521, USA

## Abstract

DNA methylation at the C-5 position of cytosine is an important epigenetic mechanism underpinning various cellular functions, such as heterochromatin assembly, gene expression, and cell fate determination. In mammals, DNA methylation mainly occurs in the context of CpG dinucleotide contexts. Establishment and maintenance of mammalian DNA methylation is orchestrated by two groups of functionally distinct enzymes: *de novo* DNA methyltransferases DNMT3A and DNMT3B and maintenance DNA methyltransferase DNMT1. For proper genomic methylation, both *de novo* and maintenance DNMTs are subjected to multilayered regulation by the chromatin environment, such as histone modifications and the methylation stiate of the CpG dinucleotide. Furthermore, DNA methylation is critically regulated by the accessory proteins of DNMTs, such as DNMT3L and DNMT3B3 for *de novo* methylation and E3 ubiquitin ligase UHRF1 for maintenance DNA methylation. Increasing structural, biochemical and cellular evidence has unveiled the intricate interplay between the conformational dynamics of DNMTs and their target specification in governing the dynamic DNA demethylation across the genome. This review focuses on recent advances in structural and functional understanding of DNMTs, emphasizing how the interplay between their intramolecular and intermolecular interactions modulates the conformational dynamics and function of the individual DNA methylation machinery, thereby shaping the dynamic DNA methylation landscape across the mammalian genome.

## INTRODUCTION

The conformational dynamics of proteins intimately impacts their biological functions, ranging from substrate accessibility and catalytic turnovers of single-modular enzymes to the functional assembly of multi-modular, multi-subunit complexes in signal transduction. In the past two decades, it has become increasingly clear that protein conformational dynamics constitutes a key molecular principle in epigenetic regulation: through alternative protein interaction mechanisms, it helps modulate the interconversion between various functional states of epigenetic complexes, thereby attaining chromatin environment-dependent activity within complex eukaryotic genomes.

Eukaryotic genome is organized into the form of chromatin, with nucleosome as the basic unit. The structure and function of chromatin is critically regulated by epigenetic mechanisms,^1,2^ i.e., the heritable changes in gene expression that occur beyond the change in the underlying DNA sequence.^1^ With the identification of the increasing number of chromatin modifiers and effectors,^3,4^ the field of epigenetic research has undergone rapid expansion in mechanistic investigation, spanning from covalent chromatin modifications, notably histone modifications and DNA methylation, to non-coding RNAs.^5,6^ One of the important themes in epigenetic regulation lies in its intricate interplay with environment and genome complexities.^7^ Molecular understanding of the functional regulation of epigenetic machinery is essential for illustrating the epigenetic programming and reprogramming process in development.

DNA methylation at the C-5 position of cytosines provides an important mechanism for silencing of transposable elements,^8,9^ genomic imprinting,^10^ and X-chromosome inactivation.^11^ In mammals, DNA methylation mainly occurs in the context of CpG dinucleotide context,^12^ catalyzed by two groups of enzymes, namely, *de novo* DNA methyltransferases and maintenance DNA methyltransferases.^13^ The *de novo* DNA methyltransferases—DNMT3A, DNMT3B, and the rodent-specific DNMT3C—establish methylation patterns during embryogenesis and gametogenesis.^14,15^ Subsequently, the DNA methylation patterns are mainly propagated by the maintenance DNA methyltransferase DNMT1, the activity of which is strongly coupled with DNA replication.^13^ The DNA methylation activities of DNMT1 and DNMT3s are further regulated by various chromatin factors, such as E3 ubiquitin ligase UHRF1 [ubiquitin-like with plant homeodomain (PHD) and RING finger domains 1] that functions to recruit DNMT1 to replicating heterochromatin through ubiquitylation of histone H3,^16–18^ DNMT3A-like (DNMT3L) that stabilizes and recruits DNMT3A to imprinted regions,^19–22^ DNMT3B isoform 3 (DNMT3B3) that regulates the activity of DNMT3B in embryonic stem (ES) cells^23^ and DNMT3A in differentiated cells.^24^ In addition, state-specific recognition of certain histone modifications and DNA sequences by the regulatory domains of DNMTs plays an important role in guiding DNA methylation across various chromatin regions.^25,26^

Structural biology serves as a cornerstone tool in transforming our understanding of structure, mechanism, and functional regulation of DNA methylation machinery.^27^ In the past two decades, various structural biology approaches, such as crystallography, single-particle cryo-electron microscopy (cryo-EM) and NMR spectroscopy, integrated with biochemical assays, have revolutionized our view on the conformational dynamics of DNMTs that governs their substrate recognition, enzymatic catalysis, and chromatin targeting.^28–57^ The structures of DNMTs are multi-modular and plastic, providing a mechanism by which DNMTs can be selectively activated or inhibited in the developmental stage and chromatin environment-dependent manner, thereby ensuring proper establishment and maintenance of the DNA methylation patterns in development. In this review, I summarize recent advances in our understanding of the conformational dynamics of the multi-domain enzymes in maintenance and *de novo* DNA methylation, and how it gives rise to distinct functionalities of DNMTs under various chromatin environments.

### Conformational dynamics of DNMT1 in maintenance DNA methylation

**FIG. 1.**
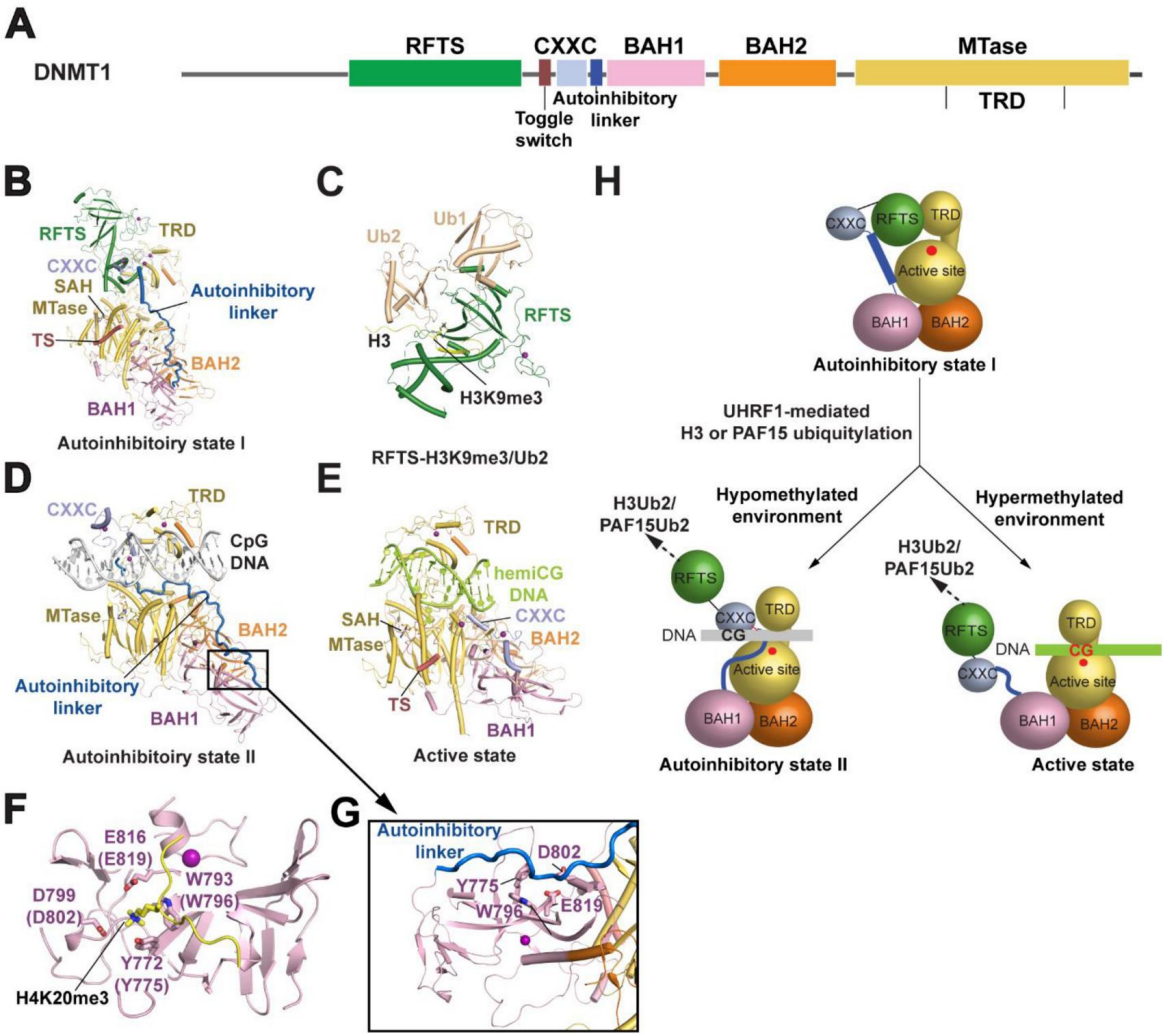
Conformational dynamics of DNMT1 in maintenance DNA methylation. (a) Domain architecture of DNMT1, with individual domains color coded. (b) Crystal structure of a C-terminal fragment (residues 351–1600) of human DNMT1 (PDB 4WXX). This structure reveals the RFTS domain-mediated autoinhibition (autoinhibitory state I). (c) Crystal structure of bovine DNMT1 RFTS domain bound to a H3K9me3 peptide and two ubiquitin molecules. (d) Crystal structure of a C-terminal fragment (residues 728–1600) of human DNMT1 in complex with unmethylated CpG DNA (PDB 3PT6). This structure reveals the CXXC domain-mediated autoinhibition (autoinhibitory state II). (e) Cryo-EM structure of a C-terminal fragment (residues 351–1616) of human DNMT1 in complex with hemimethylated CpG (hemiCG) DNA (PDB 7XI9). (f) Crystal structure of bovine DNMT1 BAH1 domain bound to a H4K20me3 peptide (PDB 7LMK), with the H4K20me3-binding residues shown in stick representation. (g) Close-up view of the DNMT1-CpG DNA complex (PDB 3PT6), highlighting the occlusion of the H4K20me3-binding site (Y775, W796, D802, and E819) by the autoinhibitory linker. (h) A model for the transition between three alternative conformational states of DNMT1: autoinhibitory state I, autoinhibitory state II and active state. Subsequent to UHRF1-mediated H3 or PAF15 ubiquitylation, the interaction of DNMT1 with H3Ub2 or PAF15Ub2 leads to release of the RFTS domain-mediated autoinhibition. Under the hypomethylated environment, DNMT1 remains in an inactive state due to the CXXC-CpG interaction that leads to blocking of the substrate access to the catalytic site. Under the hypermethylated environment, DNMT1 is fully active and engages in maintenance DNA methylation.

DNMT1 is a multi-domain protein of approximately 1600 amino acids.^58^ It contains an N-terminal disordered segment that interacts with replication-associated factors such as proliferating cellular nuclear antigen (PCNA)^59^ and DNMT1 associated protein 1 (DMAP1),^60^ followed by a replication-foci targeting sequence (RFTS) domain,^61^ a CXXC zinc finger, a pair of tandem bromo-adjacent homology (BAH) domains, and the C-terminal methyltransferase (MTase) domain^26,41,58^ [Fig. 1(a)]. The role of DNMT1 in maintenance DNA methylation is in part supported by its 3–40-fold substrate preference for hemimethylated over unmethylated CpG DNA.^62^ Still, this limited intrinsic enzymatic preference for hemimethylated DNA is insufficient to maintain the DNA methylation patterns with high fidelity, raising the long-standing question as how DNMT1 achieves the remarkable fidelity of methylation inheritance observed in mammalian genomes.^63^ As discussed below, the activity-coupled dynamic conformation of DNMT1, orchestrated by the functional interplay between the regulatory domains of DNMT1, DNA sequence, and histone modifications, promises to provide an answer to this question.

#### The RFTS domain-mediated autoinhibition: A mechanism linking maintenance DNA methylation to heterochromatic regions

The RFTS domain has long been recognized as a critical module for localizing DNMT1 to the replication fork during the S phase.^61^ Yet, a mechanistic understanding of the RFTS domain-mediated regulation was illuminated only after the structural and biochemical analyses of the mouse and human DNMT1 fragment spanning from the RFTS domain toward the C-terminal MTase domain.^43,50,64^ Notably, the structures of the RFTS-containing DNMT1 fragments revealed that the RFTS domain packs against the target recognition domain (TRD) of the catalytic domain, thereby blocking the DNA-binding interface of the latter^43,50^ [Fig. 1(b)]. Consistently, in a separate study, the fragment-based *in vitro* DNA methylation assays showed that removal of the RFTS domain led to over 600-fold increase in the DNA methylation activity.^64^ These observations therefore suggest the role of the RFTS domain in DNMT1 autoinhibition.

The functional context of the RFTS domain-mediated autoinhibition was further illuminated by the findings that the RFTS domain specifically reads dual mono-ubiquitylation of histone H3 at lysines 14, 18, and/or 23 (H3Ub2)^17,33,65^ or PCNA-associating factor 15 at lysines 15 and 24 (PAF15Ub2),^66^ both of which are a transient mark catalyzed by UHRF1.^17,33,65^ Structural and biochemical analysis of the RFTS-H3Ub2 complex revealed that a surface groove between the N-terminal and C-terminal lobes of the RFTS domain harbors the H3 N-terminal tail, while the loop segments in the N-lobe make contact with the H3K18- and K23-conjugated ubiquitin moieties, thereby attaining the specific recognition of H3Ub2.^33^ Importantly, the H3Ub2-binding interface of the RFTS domain overlaps in part with its TRD-contact interface, suggesting competitive binding of the RFTS domain with the H3Ub2 and the TRD.^33^ Indeed, structure-guided biochemical analysis reveals that the RFTS-H3Ub2 interaction led to destabilization of the RFTS–TRD interface, thereby facilitating the release of DNMT1 autoinhibition.^33^ Subsequently, we found that DNMT1's RFTS domain is a specific reader for both H3Ub2 and the heterochromatin-associated histone mark H3K9me3, providing a mechanism in fine-tuning the DNMT1 activity in H3K9me3-enriched chromatin regions^39^ [Fig. 1(c)]. Disruption of these interactions compromises DNA methylation fidelity and sensitizes cells to ionic radiation stress.^39,40^ Together, these findings establish the RFTS domain as a regulatory hub that integrates histone modifications with DNMT1 activation, a paradigm supported by molecular dynamics simulations and single-molecule fluorescence resonance energy transfer (FRET) analyses.^33,39^

#### The CXXC domain-mediated autoinhibition: A mechanism preventing DNMT1-mediated DNA methylation in a hypomethylated environment

The CXXC domain is a specific reader for unmodified CpG DNA^67^ that is present in a variety of chromatin modifiers, such as histone H3 lysine-4 methyltransferase mixed-lineage leukemia 1 (MLL1),^68^ histone lysine demethylase 2A or 2B (KDM2A/2B),^69^ ten–eleven translocation methylcytosine dioxygenase 1 or 3 (Tet1/3),^70,71^ CXXC finger protein 1 (CFP1),^72^ and DNMT1.^41^ Most of these CXXC domain-containing proteins engage the CpG islands (CGIs) for epigenetic regulation.^69,71,73–80^ For instance, the CXXC domains in MLL1 and KDM2B protect the unmethylated CpG sites in the CGI-overlapping polycomb target regions from hypermethylation.^68,69^ The CXXC domain in Tet3 helps restrict its dioxygenase activity to the CGIs, thereby safeguarding the hypomethylation status of the corresponding region.^70,71^ In addition, the CXXC domain of the Tet2-interacting partner IDAX functions to regulate the activity and protein expression of Tet2.^81^ However, the role of the CXXC domain in a repressive chromatin modifier like DNMT1 has long been elusive. To address this requestion, we previously solved the crystal structure of a C-terminal fragment of DNMT1, comprising the CXXC domain, a pair of BAH domains and the MTase domain in complex with CpG DNA^41^ [Fig. 1(d)]. The structure surprisingly revealed an autoinhibitory conformation of DNMT1, in which the CXXC domain binds to the unmethylated CpG site, which in turn positions the CXXC domain, along with its downstream linker (a.k.a. autoinhibitory linker), on top of the DNA-binding site of the TRD, thereby blocking the access of the DNA to the catalytic site^41^ [Fig. 1(d)]. This structural observation prompted us to hypothesize that in a hypomethylated environment, the CXXC-CpG interaction serves to restrict the DNA methylation activity of DNMT1 toward an unmethylated CpG site, i.e., *de novo* DNA methylation activity. Indeed, removal of the CXXC domain led to substantial increase in its DNA methylation activity on unmethylated CpG-containing DNA.^41^ This notion is further supported by a recent study showing that the CXXC-CpG interaction contributes to the substrate specificity of DNMT1 during DNA association, but not during the processing methylation mode.^82^ More recently, structural study of another C-terminal fragment of DNMT1, spanning from the RFTS domain to the MTase domain, in complex with a hemimethylated CpG substrate reveals that in an active state of the DNMT1–DNA complex, the CXXC domain moves from the TRD-proximal site to the surface of the BAH1 domain^35^ [Fig. 1(e)]. As a result, the active site of DNMT1 is fully exposed for DNA access.^35^

Structural comparison of the DNMT1 molecule under the RFTS- and CXXC-mediated autoinhibition reveals that the transition between the two alternative autoinhibitory states is accompanied by ∼30-Å lateral repositioning of the CXXC domain, as well as a helix-to-loop conformational transition of the autoinhibitory linker, indicative of a large conformational change.^50^ Nevertheless, the autoinhibitory linker, through alternative intramolecular interactions with the RFTS and the MTase domains, is instrumental in stabilizing both autoinhibitory states. It is worth noting that structural analysis of the apo-form DNMT1 (PDB 4WXX) and the DNMT1-CpG DNA complex (PDB 3PT6) both reveals that the autoinhibitory linker further traverses across the BAH1 domain, leading to the occlusion of the H4K20me2/3-binding pocket of the BAH1 domain [Figs. 1(f) and 1(g)]. These observations not only link the BAH1-H4K20me2/3 readout to DNMT1 activation, a mechanism critical for the DNA methylation maintenance of LINE1 transposons,^40^ but also testified to a multifaceted role of autoinhibition in the functional regulation of DNMT1.^41,50^

Together, the structure–function characterizations of DNMT1 thus far highlight the importance of the conformational dynamics of DNMT1 in maintenance DNA methylation. These studies suggest that the multi-modularity of DNMT1 provides a basis for its intricate interplay with chromatin environment (DNA sequence and histone modifications), which might be necessary for tailoring the DNMT1 activity toward the hypermethylated environment of heterochromatin [Fig. 1(h)].

### Conformational dynamics of UHRF1 in maintenance DNA methylation

**FIG. 2.**
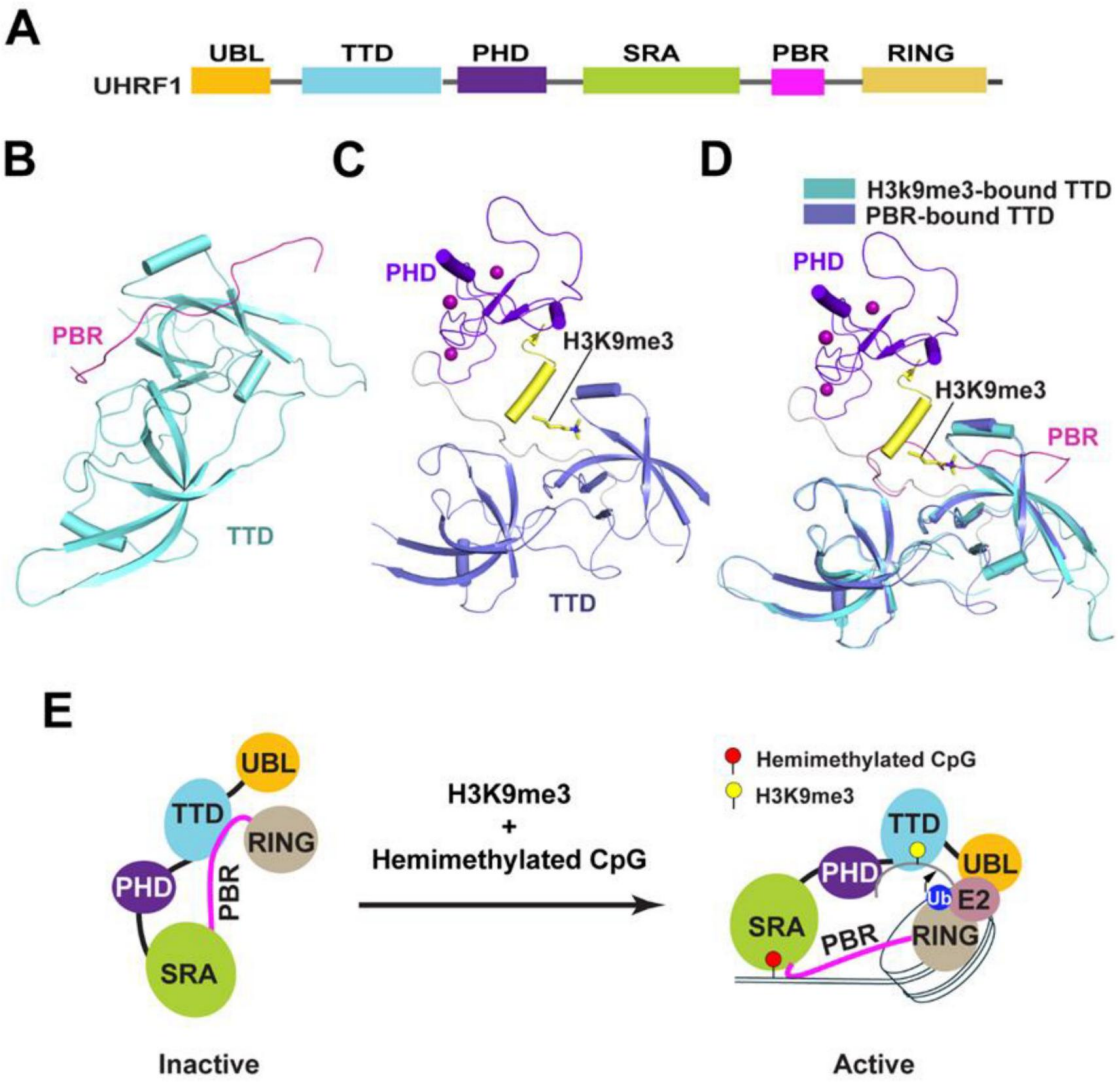
Conformational dynamics of UHRF1 in regulating maintenance DNA methylation. (a) Domain architecture of UHRF1, with individual domains color coded. (b) Crystal structure of UHRF1 TTD domain in complex with the PBR peptide (PDB 6B9M). (c) Crystal structure of UHRF1 PhD-TTD dual domain in complex with a H3K9me3 peptide (PDB 4GY5). (d) Structural overlay of the UHRF1 PHD-TTD in complex with the H3K9me3 peptide (PDB 4GY5) and the UHRF1 TTD-PBR (PDB 6B9M) complex, highlighting the steric clash between the PBR and the H3K9me3 peptide. (e) A model for the functional interplay between the intramolecular TTD-PBR interaction of UHRF1 and the intermolecular interaction of UHRF1 with other chromatin factors, such as H3K9me3 and hemimethylated CpG DNA. The TTD-PBR interaction leads to a closed conformation of UHRF1, corresponding to an E3 ubiquitin ligase-inactive state, while the interaction of UHRF1 with H3K9me3 or hemimethylated CpG DNA leads to an open conformation and enzymatically active state.

UHRF1 is a multi-domain E3 ubiquitin ligase that acts as a critical cofactor for DNMT1-mediated maintenance DNA methylation.^16,18^ It contains five conserved domains: an N-terminal ubiquitin-like (UBL) domain, a tandem Tudor domain (TTD), a plant homeodomain (PHD) finger, a SET- and RING-associated (SRA) domain, and a C-terminal RING finger [Fig. 2(a)]. The UBL domain was shown to regulate UHRF1-mediated ubiquitylation through interaction with the E2 ubiquitin ligase.^83,84^ The TTD and PHD contribute to the chromatin association of UHRF1 through recognition of the histone H3 tail—H3K9me2/3, preferentially in combination with H3K4me1,^85^ and unmodified H3R2, respectively.^46,86–92^ In addition, a polybasic repeat (PBR) located in the linker between the SRA and RING domains plays a role in protein interaction with various functional partners, such as USP7 and DNMT1.^30,45,47^ The SRA domain specifically recognizes hemimethylated CpG dinucleotides.^93–95^ It has been demonstrated that the enrichment of DNMT1 in the replicating heterochromatin was lost in *Uhrf1^−^*^/*−*^ mouse embryonic stem (ES) cells, whereas expressing a Myc-tagged mouse Uhrf1 protein in *Uhrf1^−^*^/*−*^ cells rescued the DNMT1 localization pattern.^16,18^ Consistently, *Uhrf1^−^*^/*−*^ mouse ES cells show a significantly hypomethylated genome landscape that phenocopies the DNMT1 knockout cells.^16,18^ Understanding how the individual domains of UHRF1 cooperate to regulate its chromatin association and ubiquitin ligase activity is key for mechanistic elucidation of maintenance DNA methylation.

#### The TTD domain-mediated autoinhibition: A mechanism potentiating the UHRF1-mediated H3 ubiquitylation in replicating heterochromatic foci

An early biochemical study indicates that UHRF1 may adopt two alternative states for chromatin association: in H3K9me0-bound state, the intramolecular interaction of the TTD domain with a C-terminal PBR leads to the occlusion of the H3K9me3-binding site of the former. In the H3K9me3-bound state, the TTD-PBR interaction is disrupted; instead, the TTD-PHD dual domain forms a platform for multivalent interaction with H3K9me3 and H3R2.^96^ In support of this notion, subsequent structural and biochemical evidence indicates that the interaction of the UHRF1 PBR with USP7 or hemimethylated CpG DNA promotes the heterochromatin association^45,47,97^ and the E3 ubiquitin ligase activity of UHRF1.^45,98^ Along the line, structural studies of the UHRF1 TTD domain in complex with the PBR peptides from others and us provided atomic details for the intramolecular interaction of UHRF1 and its interplay with other functional regulators^31,47^ [Fig. 2(b)]. Importantly, we observed that the PBR docks onto the surface of the TTD, occluding the aromatic cage that would normally bind the H3K9me3 mark^31^ [Fig. 2(d)]. Consistent with the structural study, our isothermal titration calorimetry (ITC) binding assays showed that the TTD-PBR interaction competes against the TTD-H3K9me3 interaction, confirming that the PBR-TTD intramolecular interaction inhibits the histone‐reader function of the TTD domain. In addition, our FRET analysis of full-length UHRF carrying an N-terminal CFP tag and a C-terminal YFP tag, combined with *in vitro* peptide pull-down assays, showed that the conformation of UHRF1 switches from a “closed” state (TTD bound to PBR) to an “open” state (PBR relocated, TTD free to engage H3K9me3) upon binding to hemimethylated DNA or USP7.^31^

Together, these studies demonstrated that the autoinhibitory conformation of UHRF1 serves as a checkpoint for the epigenetic state of the chromatin environment, ensuring proper control of UHRF1 activity across various genomic regions [Fig. 2(e)].

### Conformational dynamics of DNMT3A and DNMT3B in *de novo* DNA methylation

**FIG. 3.**
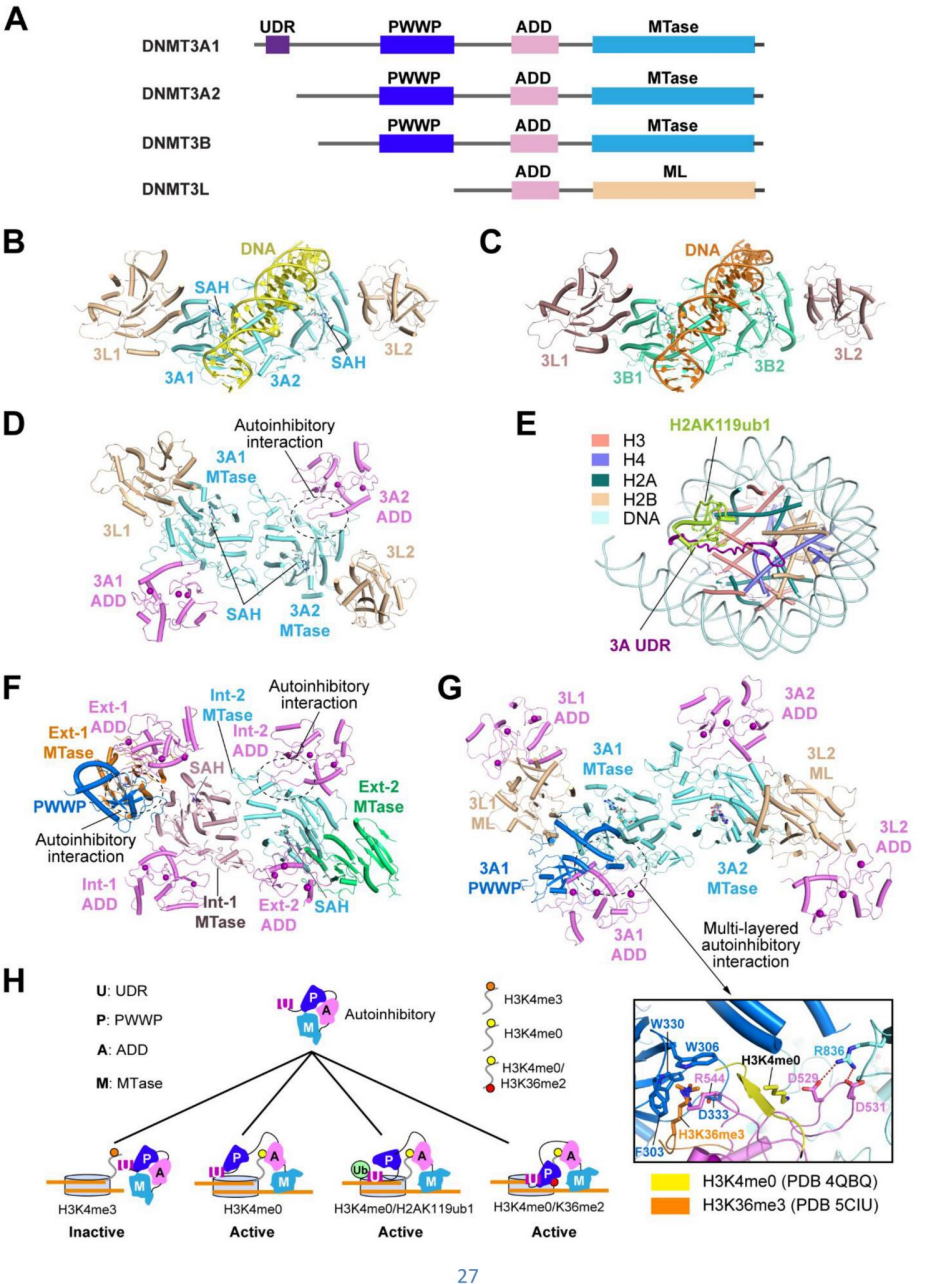
Conformational dynamics of DNMT3A and DNMT3B in *de novo* DNA methylation. (a) Domain architecture of DNMT3A isoform 1 (DNMT3A1) and 2 (DNMT3A2), DNMT3B and DNMT3L, with individual domains color coded. (b) and (c) Crystal structures of DNMT3A^MTase^–DNMT3L^ML^–CpG DNA (PDB 5YX2) (b) and DNMT3B^MTase^–DNMT3L^ML^–CpG DNA (PDB 6U8P) (c) complexes. (d) Crystal structure of the autoinhibitory DNMT3A^ADD−MTase^–DNMT3L^ML^ complex (PDB 4U7P). The key residues involved in the autoinhibitory interaction between the ADD and MTase domains of DNMT3A are shown in stick representation in the expanded view. (e) Cryo-EM structure of DNMT3A1 UDR in complex with H2AK119ub1-modified nucleosome (PDB 8U5H). (f) Cryo-EM structure of DNMT3B homo-tetramer (PDB 8EIH). The domain interface for the autoinhibitory interaction between the ADD and MTase domains of the DNMT3B inner subunit and for the autoinhibitory interaction between the PWWP, ADD and MTase domains of the DNMT3B outer subunit are marked by dashed circle, respectively. (g) Cryo-EM structure of DNMT3A2–DNMT3L complex (PDB 9PRW). The domain interface for the PWWP-ADD-MTase autoinhibitory interactions is shown in the expanded view. The side chains of the H3K36me3-binding residues (F303, W306, W330, and D333) and the H3K4me0-binding residues (D529 and D531) are shown in representation. The electrostatic interaction between ADD residues D529 and D531 and the target recognition domain residue R836 is shown as dashed line. Note that the PWWP-ADD interaction leads to occlusion of the H3K36me2/3-binding site, while the ADD-MTase interaction leads to occlusion of the H3K4me0-binding site. (h) A model for the autoinhibitory regulation of DNMT3A, which functions to differentially control the DNA methylation activity of DNMT3A at various chromatin regions.

DNMT3A and DNMT3B are responsible for establishing DNA methylation patterns *de novo* in development.^15^ DNMT3A is important for establishing the DNA methylation at major satellite repeats, allele-specific imprinting loci and intergenic repeats during gametogenesis,^21,99,100^ whereas DNMT3B mediates the minor satellite repeat and gene body methylation during early embryonic development.^15,99,101^ Both DNMT3A and DNMT3B contain a C-terminal MTase domain preceded by a Pro-Trp-Trp-Pro (PWWP) domain and an ATRX–DNMT3–DNMT3L (ADD) domain [Fig. 3(a)]. The PWWP domain recognizes the H3K36me2/3 mark and DNA,^51,100,102–104^ while the ADD domain recognizes the H3K4me0 mark.^38,105^ In addition, DNMT3A isoform 1 (DNMT3A1) contains an additional N-terminal tail that binds to the nucleosome core marked by H2AK119ub1.^48,106–109^ The DNMT3A/DNMT3B-mediated DNA methylation is further regulated by DNMT3L and DNMT3B3, which associate with DNMT3A or DNMT3B into a heterotetramer to modulate the *de novo* DNA methylation activity at various developmental stages.^19–22,110,111^ Dysregulation of DNMT3A and DNMT3B have been linked to various human diseases, with DNMT3A mutation associated with acute myeloid leukemia (AML),^112^ myelodysplastic syndrome (MDS),^113^ paraganglioma,^114^ microcephalic dwarfism^115^ and Tatton–Brown–Raman syndrome (TRBS),^116^ and DNMT3B mutation linked to the immunodeficiency, centromeric instability, facial anomalies (ICF) syndrome.^15,117–119^ How the *de novo* DNA methyltransferases DNMT3A and DNMT3B are regulated for proper methylation on their target chromatin regions is one of the long-standing questions in epigenetics.

#### Mechanistic basis of DNMT3A- and DNMT3B-mediated DNA methylation

An early structural study of the DNMT3A MTase domain in complex with the methyltransferase-like (ML) domain of DNMT3L reveals a 3L-3A-3A-3L assembly, in which DNMT3A homodimerizes via a hydrophilic interface mediated by reciprocal arginine-aspartate salt bridges (a.k.a. RD interface), while each of the DNMT3A subunits is further associated with one DNMT3L ML domain through the hydrophobic interface mediated by the stacking interactions of a cluster of phenylalanine residues (FF interface).^34^ Subsequent structural studies of the DNMT3B–DNMT3L, DNMT3A–DNMT3B3, DNMT3A homo-oligomer and DNMT3B homo-oligomer reveals a similar assembly mechanism.^29,36,37,111,120^ For DNMT3A and DNMT3B homotetramers, the external subunits are structurally disordered in the TRD and the cofactor-binding sites, mirroring the structures of DNMT3L and DNMT3B3, in which the corresponding sequences were lost during evolution.^36,37^ Such a tetrameric assembly of DNMT3A/3B on one hand provides an extensive DNA-binding interface; on the other hand, allows for multivalent chromatin association of DNMT3A and DNMT3B.

Structural insights into the DNMT3A- and DNMT3B-mediated *de novo* DNA methylation were gained from the structural studies of the DNA-bound forms of the DNMT3A–DNMT3L and DNMT3B–DNMT3L complexes^28,29,44,120^ [Figs. 3(b) and 3(c)]. Through incorporation of zebularine, a cytosine analog, into the DNA substrate, we were the first to capture a reaction intermediate state in which the catalytic cysteine of DNMT3A or DNMT3B forms a covalent adduct with zebularine.^28,29,44,120^ The structures of these complexes reveal that the substrate recognition of DNMT3A and DNMT3B was mediated by three distinct structural regions: the catalytic loop, a loop of the target recognition domain (TRD loop), and the helix at the homodimeric RD interface of DNMT3A (RD-helix).^28,29,44,120^ Notably, the catalytic loop invades into the minor groove to interact with both DNA strands, contributing to the stabilization of orphan guanine that is otherwise paired with the target cytosine, the CpG guanine on the target strand, as well as the flipped zebularine in the active site.^28,29,44,120^ The TRD loop forms a hydrogen bond with the CpG guanine on the target strand from the major groove, which contributes to the CpG-specificity of DNMT3A/3B as well as their distinct flanking sequence preference.^28,29,44,120^ In addition, the RD-helix of DNMT3A/3B interacts with the DNA backbone, contributing to the substrate association of DNMT3A/3B.^28,29,44,120^

#### Conformational dynamics of the ADD domain links histone H3K4me0 readout to *de novo* DNA methylation

The functional importance of the DNMT3A ADD domain is highlighted by the observation that disruption of its H3K4me0-binding site led to mosaic loss of DNA methylation in maternal imprinting control regions (ICRs) of mouse oocytes.^121^ Aside from the H3K4me0-binding activity, the DNMT3A ADD domain was shown to directly interact with the catalytic site of the MTase domain in the crystal structure of the DNMT3A ADD-MTase fragment complexed with the DNMT3L ML domain, leading to occlusion of the DNA-binding site of the MTase domain as well as the H3K4me0-binding site of the ADD domain^32^ [Fig. 3(d)]. Upon binding to a H3K4me0 peptide, the DNMT3A ADD domain is repositioned away from the active site of the MTase domain, thereby allowing the DNA access.^32^ Therefore, these structural observations reveal an autoinhibitory mechanism of DNMT3A that couples the H3K4me0 binding with its enzymatic activation, providing an explanation to an earlier study that the H3K4me0 mark allosterically stimulates DNMT3A activity.^122^ The caveat of these observations is that formation of the DNMT3A–DNMT3L crystals is in part attributed to the ADD domain-mediated packing interactions.^32^ In fact, our recent cryo-EM structural studies of DNMT3A2–DNMT3L and DNMT3B homo-oligomer reveal that while the ADD domains of DNMT3A and DNMT3B indeed interact with their respective MTase domains, they involve a different contact interface: instead of interacting with the catalytic site as observed in the crystal structure [Fig. 3(d)], the cryo-EM structures showed that the ADD domain interacts with the TRD loop, a separate DNA-binding site of the MTase domain^36,123^ [Fig. 3(e)]. Through molecular dynamics simulation analysis, our study further demonstrated that upon the ADD-H3K4me0 interaction, the TRD loop moves away from the ADD domain for potential DNA contact, eventually leading to DNMT3A activation.^123^ These distinctive structural observations highlight the importance of structural studies of proteins under their native states.

#### The PWWP domain-mediated autoinhibition ensures optimal activity of DNMT3A/3B at H3K36me2/3-enriched regions

The PWWP domains of DNMT3A and DNMT3B knowingly mediate their targeting to the H3K36me2-enriched regions (e.g., intergenic repeats) and H3K36me3-enriched region (e.g., gene body), respectively.^100,101,124^ In addition, the PWWP domain contributes to the recruitment of DNMT3A/3B to pericentric heterochromatin.^125^ The functional importance of the PWWP domain is further highlighted by the fact that mutation of the DNMT3B PWWP domain (e.g., S270P) has been identified in ICF syndrome,^126^ and mutation of the DNMT3A PWWP domain (e.g., W330R and D333N) causes a gain-of-function effect of DNMT3A in paraganglioma or microcephalic dwarfism.^107,114,115,127^ However, detailed structure–function characterization of the PWWP domain in functional regulation of DNMT3A and DNMT3B has long been hurdled by the dynamic conformation of this domain relative to the ADD and MTase domains.

Taking advantage of the cryo-EM approach, we recently reported the structure of the homo-oligomeric DNMT3B fragment, encompassing the region from the PWWP domain toward the MTase domain.^36^ Our study reveals that the PWWP domain of one of the DNMT3B subunits (likely the inner subunits) interacts with the ADD and MTase domains of the outer subunits, thereby blocking the DNA-binding site of the outer subunit^36^ [Fig. 3(f)]. Consistently, our biochemical analysis further demonstrated that disrupting the interaction between the PWWP and the ADD-MTase modules led to enhanced DNA methylation activity.^36^ Likewise, in another of our recent study, the structure of the DNMT3A2–DNMT3L complex revealed that the PWWP domain joins with the ADD domain to orchestrate a multi-layered DNMT3A autoinhibiton^123^ [Fig. 3(e)]. On one hand, the DNMT3A ADD domain interacts with the TRD of the MTase domain within the same subunit, leading to occlusion of the DNA-binding site of the TRD as well as the H3K4me0-binding site of the ADD domain; on the other hand, the PWWP domain interacts with the MTase and ADD domains, which not only contributes to the occlusion of the DNA-binding site in the TRD loop of the MTase domain, but also the occlusion of the H3K36me2/3-binding site of the PWWP domain^123^ [Fig. 3(e)]. Our structure-guided biochemical analysis further demonstrates that the PWWP domain-mediated autoinhibition serves to couple the H3K36me2/3 with DNMT3A activation, thereby ensuring optimal DNA methylation activity in H3K36me2/3-enriched regions. Together, these observations suggest that the PWWP domain of DNMT3A or DNMT3B not only plays a role in their chromatin targeting but also regulates their functional assembly and DNA methylation activity.^36,123^

#### The UDR-H2AK119Ub1 interaction recruits DNMT3A1 to the polycomb targets for developmental stage-specific gene silencing

Two recent studies have identified the N-terminal UDR of DNMT3A1 as a specific reader for H2AK119Ub1.^106,107^ The UDR-H2AK119Ub1 interaction is responsible the recruitment of DNMT3A1 to polycomb targets, thereby regulating the expression of the bivalent neurodevelopmental genes.^106^ Along the line, the structural study of DNMT3A1 UDR in complex with H2AK119Ub1-modified nucleosome from us and others revealed extensive interaction of DNMT3A1 UDR with the nucleosome surface, spanning the H2A–H2B acidic patch, H3 homodimeric interface, nucleosomal DNA, and the H2AK119Ub1 moiety^48,108,109^ [Fig. 3(g)]. The DNMT3A1 UDR-H2AK119Ub1 therefore adds another layer of regulation in *de novo* DNA methylation.

Together, these specific interactions between the regulatory domains of DNMT3A or DNMT3B and epigenetically modified nucleosomes provide a mechanism to direct the optimal activity of DNMT3A/3B toward the regions enriched with H2K119Ub1, H3K4me0 and/or H3K36me2/3, thereby attaining target specificity of *de novo* DNA methylation [Fig. 3(h)].

## CONCLUSION

The structure and function of mammalian DNA methylation machinery is subject to tight spatial–temporal regulation for proper transcriptional programming and genome stabilization in development. The interplay between the conformational dynamics of DNA methylation machinery and region-specific chromatin cues, such as repressive histone modifications, DNA sequence composition, and higher-order nuclear organization provides an important regulatory mechanism for specifying the chromatin region-dependent DNA methylation patterns. For *de novo* DNA methylation, our current structural and biochemical evidence has pointed to a multi-layered autoinhibition mechanism for DNMT3A and DNMT3B. Through coupling of the DNA methylation activities of DNMT3A/3B with their interaction with H3K4me0 and H3K36me2/3 marks, these mechanisms help limit the DNMT3A- and DNMT3B-mediated DNA methylation to their target regions, thereby ensuring proper DNA methylation establishment in development. For maintenance DNA methylation, the conformational dynamics of UHRF1 between alternative functional states provides a mechanism in coupling its activity with replicating heterochromatin (e.g., H3K9me3, hemimethylated CpG DNA, etc.), while the RFTS domain- and the CXXC domain-mediated autoinhibition of DNMT1 offer another layer of regulation to ensure high-fidelity maintenance of DNA methylation at UHRF1-targeted regions, rather than hypomethylated environment.

Despite the advance in mechanistic understanding of the DNA methylation regulation, how DNMTs crosstalk with the chromatin environment to adapt to genome complexity remains a long-standing question. In this regard, the rapid development of single-particle cryo-EM, cryo-electron tomography and single-molecule fluorescence methods place us in an unprecedented position to reveal how the DNA methylation enzymes transit between various functional states in living cells. Such insights will not only illuminate the fundamental principles of *de novo* and maintenance methylation but also inform the development of targeted therapies for cancer and epigenetic disorders.

## AUTHOR'S NOTE

This special issue offers a unique opportunity to celebrate Dr. John L. Markley's remarkable contribution to the field of biochemistry, structural biology and NMR spectroscopy. I had the privilege of working with John for ten years, first as one of his graduate students and then as a staff scientist in the center for eukaryotic structural genomics. During that time, I had been intrigued by his strong vision in NMR spectroscopy and scientific rigor in biochemical research. Thanks to his guidance and support, I had the opportunity to characterize the structure, dynamics and function of proteins from diverse biological pathways.^128–150^ For instance, we have characterized the protein dynamics at various extents and time scales, from local prolyl cis–trans isomerization of structurally compact proteins to global disorder of intrinsically disordered proteins.^131,142,147^ We reported that functionally relevant secondary and tertiary structures, despite being transient, are encoded in the native state of intrinsically disordered protein PDEγ, providing direct evidence to the conformational selection theory of protein interaction.^147^ Dr. John L. Markley's great scientific contribution is further reflected by his superior mentorship. He treated every trainee with respect and care, provided persistent support for us to explore the uncharted area of life science, which greatly shaped our career as a biochemist and educator.

## Data Availability

The data that support the findings of this study are publically available.
